# Upward Altitudinal Shifts in Habitat Suitability of Mountain Vipers since the Last Glacial Maximum

**DOI:** 10.1371/journal.pone.0138087

**Published:** 2015-09-14

**Authors:** Masoud Yousefi, Mohsen Ahmadi, Elham Nourani, Roozbeh Behrooz, Mehdi Rajabizadeh, Philippe Geniez, Mohammad Kaboli

**Affiliations:** 1 Department of Environmental Sciences, Faculty of Natural Resources, University of Tehran, Tehran, Iran; 2 Department of Natural Resources, Isfahan University of Technology, Isfahan, Iran; 3 Graduate School of Fisheries Science and Environmental Studies, Nagasaki University, Nagasaki, Japan; 4 CEFE UMR 5175, CNRS—Université de Montpellier—Université Paul-Valéry Montpellier–EPHE, laboratoire Biogéographie et écologie des vertébrés, 1919 route de Mende, 34293 Montpellier cedex 5, France; 5 Evolutionary Morphology of Vertebrates, Ghent University, Ghent, Belgium; 6 Department of Biodiversity, Institute of Science and High Technology and Environmental Sciences, Graduate University of Advanced Technology, Kerman, Iran; 7 Iranian Plateau Herpetology Research Group (IPHRG), Faculty of Science, Razi University, Kermanshah, Iran; State Natural History Museum, GERMANY

## Abstract

We determined the effects of past and future climate changes on the distribution of the *Montivipera raddei* species complex (MRC) that contains rare and endangered viper species limited to Iran, Turkey and Armenia. We also investigated the current distribution of MRC to locate unidentified isolated populations as well as to evaluate the effectiveness of the current network of protected areas for their conservation. Present distribution of MRC was modeled based on ecological variables and model performance was evaluated by field visits. Some individuals at the newly identified populations showed uncommon morphological characteristics. The distribution map of MRC derived through modeling was then compared with the distribution of protected areas in the region. We estimated the effectiveness of the current protected area network to be 10%, which would be sufficient for conserving this group of species, provided adequate management policies and practices are employed. We further modeled the distribution of MRC in the past (21,000 years ago) and under two scenarios in the future (to 2070). These models indicated that climatic changes probably have been responsible for an upward shift in suitable habitats of MRC since the Last Glacial Maximum, leading to isolation of allopatric populations. Distribution will probably become much more restricted in the future as a result of the current rate of global warming. We conclude that climate change most likely played a major role in determining the distribution pattern of MRC, restricting allopatric populations to mountaintops due to habitat alterations. This long-term isolation has facilitated unique local adaptations among MRC populations, which requires further investigation. The suitable habitat patches identified through modeling constitute optimized solutions for inclusion in the network of protected areas in the region.

## Introduction

Global climate change is responsible for large-scale alterations in geographic distributions of animal and plant species worldwide [1,2]. The magnitude of such changes is expected to increase with latitude, putting mountain-dwelling species especially at risk in the face of future climatic changes [1]. Climate-induced habitat alterations are particularly severe for reptiles [3–5]. In fact, occurrence of reptiles has been highly impacted by climatic changes throughout history, mainly due to their limited dispersal ability [6].

In general, current geographic distributions of taxa can be explained by the patterns of past climate changes, especially relative to glacial periods [7,8]. The ice ages became especially severe through the Pleistocene [7] and the most recent glaciation reached a peak about 18,000–21,000 years ago [9]. In the Iranian plateau, the resulting shifts in the snowline in mountainous regions reshuffled the geographic distribution of many taxa [10].

The *Montivipera raddei* species complex (MRC) contains rare species with small allopatric populations [11–13] that are distributed in highland habitats of Iran-Anatolian plateau and the lesser Caucasus Mountains [14]. Following Rajabizadeh (2013), MRC consists of four taxa: *Montivipera kuhrangica* [11], *M*. *latifii* [15], *M*. *raddei raddei* [16], and *M*. *raddei albicornuta* [17]. The whole complex faces severe threats that make it highly vulnerable to extinction [11,12,18]. Habitat loss is indeed the major threat, especially to *M*. *kuhrangica*, *M*. *r*. *albicornuta*, and *M*. *latifii*, which are endemic to Iran and exceptionally rare [11,19]. Illegal collection-especially of *M*. *r*. *albicornuta* and *M*. *latifii*- for antivenin production [20] is also taking a toll on these vulnerable species.

The distribution of MRC has not been thoroughly investigated and factors limiting its distribution are not understood. It is known however, that MRC populations appear in small isolated patches [11,12,21]. The recent identification of *M*. *kuhrangica*, as the most southern distribution among this complex in Zagros Mountains [11] indicates the possibility that within the matrix of seemingly unsuitable habitats for MRC, patches of suitable habitat occupied by isolated and relict populations have remained unidentified.

Ecological Niche Models (ENMs) are fundamental tools for conservation and management of species [22,23], especially those that are rare, poorly documented [24], and are consequently in higher risk of extinction [25]. Species distribution modeling increases our knowledge on distribution of species, highlights factors that are most important in shaping their distribution, and aids to make predictions about their potential distribution in the future [26–28]. Comparing ENMs with the current system of nature reserves can have great implications for conservation of threatened species.

The global climate has become considerably warmer since the Last Glacial Maximum 21 kyr BP (21,000 years before present) [29]. We therefore hypothesized that the current patchy distribution of the MRC is a response to this climatic change which has limited these mountain-dwelling species to high elevations. Due to their dependence on cool climate [30], we further hypothesized that with the current rate of global climate warming, populations of this species complex will lose much of their currently suitable habitat and will be forced to move to even higher elevations to survive. To test these hypotheses, we used niche-based models and GIS to investigate the historical biogeography of MRC, as well as its future distribution in the face of two climate change scenarios. We also modeled the present distribution of MRC to identify unknown localities and assess the effectiveness of the protected area network in the region for conserving this species complex.

## Materials and Methods

### Predictor variables

Explanatory variables were prepared for two modeling approaches; a general model to predict the current potential distribution of MRC, and climate-only models to evaluate possible changes on bioclimatic niche under climate change scenarios. For the general model, we used three categories of eco-geographic factors, including climate, topography and land-cover (Table 1) that have a direct interaction with the species and were chosen based on ecological theory [31]. Climate variables were obtained from WorldClim dataset [32] as interpolated climate data layers created by collecting large amounts of weather station data. We used climatic variables as the most important factors describing the species thermal tolerance as well as water availability throughout the year, temperature and precipitation in May, when vipers wake up from hibernation in our study area.

**Table 1:** Eco-geographic factors used for modeling the distribution of *Montivipera raddei* species complex in Iran, Turkey and Armenia. Variables in bold type were used in climate change scenarios and were prepared with 2.5 arc-second resolution.

Variable	Description (abbreviation)	Unit
Topographic	Altitude: Elevation above sea level (altitude)	m
	Slope steepness (slope)	%
	Solar Radiation Index (sri)	WH/m^2^ year
Cover	Distance to rainfed croplands (crop_dis)	Degrees
	Distance to mosaic vegetation/cropland (crop_veg_dis)	Degrees
	Distance to broadleaved deciduous forest /woodland (forest_dis)	Degrees
	Distance to mosaic forest or shrubland / grassland (frst_shrb_dis)	Degrees
	Distance to mosaic grassland (50–70%) / forest or shrubland (gras_shrb_dis)	Degrees
	Distance to closed to open (>15%) shrubland (shrub_dis)	Degrees
	Distance to closed to open herbaceous vegetation (herb_dis)	Degrees
	Distance to sparse (<15%) vegetation (sprs_veg_dis)	Degrees
Bioclimatic	**Annual precipitation (anulprc)**	mm
	**Precipitation in the driest month (driest)**	mm
	**Precipitation in the wettest month (wettest)**	mm
	**Precipitation seasonality (prcseas)**	adimensional
	**Annual mean temperature (anulmeantmp)**	°C
	**Minimum temperature of coldest month (coldest)**	°C
	**Maximum temperature of warmest month (warmest)**	°C
	**Temperature seasonality (tmpseas)**	adimensional
	Mean temperature of May (tmean5)	°C
	Maximum temperature of May (tmax5)	°C
	Minimum temperature of May (tmin5)	°C
	Precipitation in May (prc5)	mm

Because of the high autocorrelation between climatic variables, we extracted values for these variables from occurrence points of the species and screened them to low-correlated (*r* < 0.8) variables. Using the Shuttle Radar Topography Mission (SRTM) elevation model, three topographic explanatory variables were compiled: elevation and slope as the most important factors describing topographic context, as well as Solar Radiation Index (SRI), calculated for a period between April to June to depict total amount of incoming solar insolation (WH/m^2^ year). We considered SRI because the regarded period coincides with MRC post-hibernation, when receiving thermal energy is an important factor affecting these species. SRI was calculated in ArcGIS 9.3 Spatial Analyst Tools using the Digital Elevation Model (DEM) of the study area. For land-cover data we used cover types from Globcover v. 2.1 map [33] as a global dataset, which contains 63 cover types (including 19 types for our study area) based on the standard UN Land Cover Classification System (LCCS). We extracted cover types that described the natural background of the landscape. Moreover, to provide continuity, Euclidian distance to nearest cover patch was calculated in ArcGIS 9.3. All eco-geographic variables were prepared with the finest resolution that WorldClim dataset allowed (approximately 1 × 1 km precision).

To assess the possible bio-geographical changes in the climatic niche of the MRC, we traced the distribution of the complex over the Last Glacial Maximum (LGM) (∼ 21 kyr BP), present and future (average for 2061–2080). Herein, we developed an ENM for the present climate conditions by using 2.5 arc-second variables and subsequently projected it onto the past and future conditions. The bioclimatic data for the LGM were developed and mapped by Paleoclimate Modeling Intercomparison Project Phase II (PMIP2) based on two different global circulation models, GCM: the Community Climate System Model (CCSM), [34]), and the Model for Interdisciplinary Research on Climate (MIROC), [35]. Furthermore, distribution of MRC under possible future climate conditions was assessed for the year 2070 (on average for 2061–2080). We used the output from the general circulation model CCSM4 from the Intergovernmental Panel on Climate Change (IPCC) 4th Assessment. Based on different inputs of greenhouse gas emission drivers (i.e. population and economic growth, technological choices), land use changes, environmental policy options and adaptation processes [36], the IPCC recommended four representative concentration pathways (RCPs) which are part of the IPCC fifth assessment report finalized in 2014. Each pathway is defined by a radiative forcing value which describes the change in the amount of energy entering the atmosphere and the quantity that is reflected back, and is expressed in Watts per square meter of surface (W/m2). The radiative forcing levels estimated to be reached by the end of the century include 2.6, 4.5, 6.0 and 8.5W/m2 [37]. In this study we considered minimum and maximum values of radiative forcing levels including 2.6 and 8.5 W/m2 that describe a mitigation scenario and high emission scenario, respectively. All bioclimatic variables for the last glacial period, as well as present and future conditions were downloaded from WorldClim dataset (Table 1).

### Distribution modeling

Because of the cryptic nature of the species concerned and shortage of information on their ecology and distribution range, a set of occurrence data from 46 point localities for MRC was compiled based on data obtained from Herp-Net database as well as field surveys conducted by the authors in several years within Iran. Our fieldwork included searching suitable habitats for vipers and recording any observations. Point localities that we obtained cover the entire natural distribution area of the MRC along highland territories of Iran, Turkey and Armenia. Ecological niche modeling was performed using maximum entropy (Maxent) algorithm [38]. We selected Maxent algorithm because it has been proven to perform better than other presence-only methods (ENFA, Domain). Because of its generative algorithm in comparison with GLM/GAMs discrimination approaches, it gives better predictions when the amount of training data is small [38,39]. Hence in the case of MRC, given the inherently limited number of known populations, Maxent was used to predict areas with potential distribution. Maximum entropy, working based upon statistical mechanics and information theory, states that a probability distribution with maximum entropy (closest to uniform), subject to known constraints, is the best approximation of an unknown distribution of species. The constraints are described by the expected values of the distribution, which is gained from a set of species presence locations [38,39]. Some advantages of Maxent include requiring presence-only data and determining probability distribution in the multivariate space. Moreover, unlike regression-based techniques, Maxent incorporates the complicated interactions between predictor variables [39,40], and is accordingly less liable to autocorrelation effects than other methods.

The training area of an ENM preferably comprises environmental conditions that are accessible for the target species [41]. In this study, for the climate change evaluations we restricted the training range to an area defined by a circular buffer of 200 km enclosing all species records, and the derived model for the present climate conditions was considered as a baseline model. Next, we projected the baseline model onto the past and future conditions. To avoid negative effects when projecting an ENM onto novel conditions outside the training range of the model [42,43], for all projections we also calculated multivariate environmental similarity surface (MESS) to represent how similar a point is to training background range of the baseline model.

To make better use of the small sample size, we applied the cross-validation method in Maxent 3.3.3, where the occurrence data was randomly split into 10 equal-sized folds, and training models were created by eliminating each fold in turn. The eliminated folds were then considered to test the performance of the training models using receiver operating characteristic (ROC) plots. We used ROC as a measure of discrimination capacity to represent the models’ ability to predict species presence and absence locations by plotting sensitivity (true positive) against 1 –specificity (false positive) [38,44] and used derived Area Under the Cure (AUC) statistic as a measure of overall fit of the models. Since ROC is a threshold-independent method, considering maximum training sensitivity plus specificity logistic thresholds–as a recommended suitability threshold [45]—we averaged omission error of cross-validated folds to evaluate classification accuracy of the ENMs. We also evaluated variable importance for all models by conducting a jackknife analysis. By performing the model repeatedly leaving out one variable at a time and computing a model using each variable alone, the importance of each variable within the models was determined.

We also generated 1000 random points within the baseline climatic niche, extracted annual mean temperature of climate change scenarios for each point, and averaged this parameter through random points. Doing so helped us get a better understanding of the changes in temperature throughout the years.

### Overlap with protected area network

Evaluating the level of conservation achieved by protection areas can be done by considering species occurrence data [46–48] or using species distribution models [49,50]. To assess the degree of protection granted to MRC in the conservation areas, we overlaid the Maxent suitability map with the map of protected areas of Iran, Turkey and Armenia. The protected area network of Iran, including the newest list of national parks, protected areas and wildlife refuges was derived from Iran Department of Environment and a shape file including protected areas of Turkey and Armenia was downloaded from *http*:*//protectedplanet*.*net*.

## Results

### Ecological niche models for MRC

Results of ecological niche modeling showed that suitable habitats of MRC are patchily distributed in higher elevations of the Zagros, Alborz, and Kopet Dagh mountains within Iran, and in Ararat and lesser Caucasus in Turkey and Armenia.

**Fig 1:**
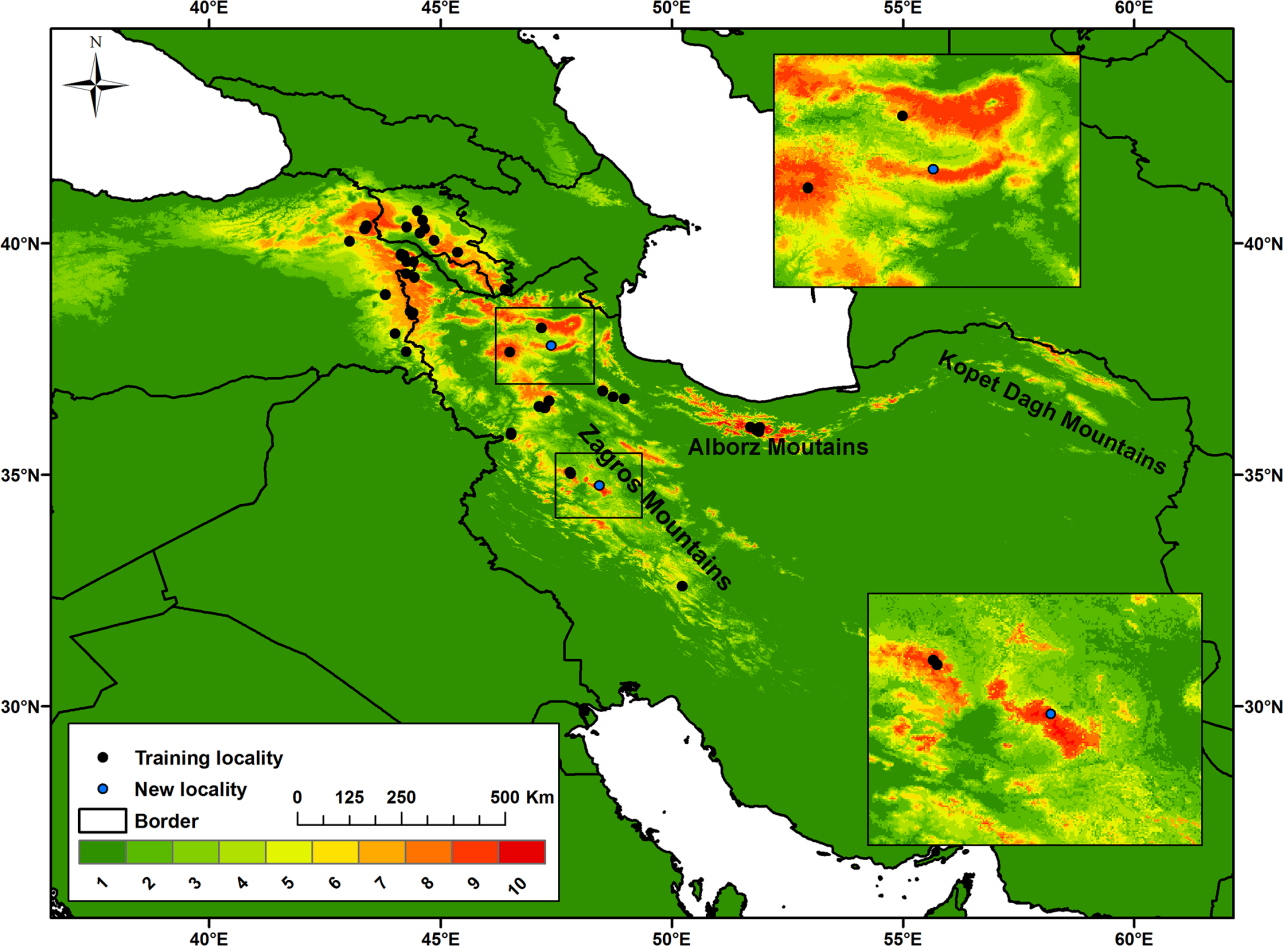
Predicted suitable and unsuitable habitats for *Montivipera raddei* species complex in Iran, Turkey and Armenia. The model includes all eco-geographic variables and was reclassified into 10 equal probability classes in which red colour shows areas with the highest probability of occurrence. Newly confirmed localities of the species are indicated with blue dots.

Interestingly, the general model with all eco-geographic variables showed that presence of MRC is predicted in areas where no previous records of occurrence exist. Reinforcing our hypothesis, during production of our paper we confirmed new localities for MRC in suitable patches in Alvand mountain in the west and Bozgoush area in the northwest of Iran (Fig 1) that were predicted in our model but not previously recorded, endorsing the good performance of the model.

The ROC plots indicated high average AUCs and low SD of AUC and omission error, reflecting the high predictive performance of our ENMs. Average AUCs for cross-validated general model and baseline climate change model was 0.969 and 0.981, respectively, where the low SD of AUCs and average omission error of the models reflected high precision (Table 2).

**Table 2:** Discrimination capacity and classification accuracy criteria for cross-validated Maxent models of *Montivipera raddei* species complex in Iran, Turkey and Armenia used for evaluating the performance of Ecological Niche Models (ENMs). Mean omission error refers to maximum training sensitivity plus specificity logistic threshold.

Model	Mean test AUC	SD of AUC	Mean omission error
General model	0.969	0.015	0.054
Baseline climate change model	0.981	0.009	0.057

### Analysis of overlap with protected areas

An overlay of the distribution map resulted from general modeling with the map of national protected areas of Iran, Turkey and Armenia indicated that 7.74% of suitable areas (with a probability of 0.33–0.66) and 2.19% of highly suitable areas (with a probability of 0.66–1) are protected by the existing protected area network.

### Main variables in the MRC distribution model

Based on results from the general model, distribution of the threatened MRC is largely determined by bioclimatic variables rather than biophysical variables. The jackknife analysis revealed that, based on the difference between regularized gains of variables, annual mean temperature, minimum and mean temperature of past hibernation period (May), minimum temperature of coldest month and distance to mosaic grassland (50–70%) / shrubland or forest were the most informative explanatory variables affecting the distribution of MRC in highland territories of the study area. However, the relative contributions of the eco-geographical variables to the general model (S1 Table) indicated that altitude (17.1%), distance to closed to open shrubland (16.6%), minimum temperature of past hibernation period (May) (14.4%), annual mean temperature (14.3%) and distance to mosaic grassland (50–70% cover) / shrubland or forest (10.5%) were the most determinant factors.

The importance of bioclimatic variables under climate change scenarios indicated that thermal tolerance is more limiting for MRC distribution than humidity. We found that annual mean temperature was the most important climatic variable followed by minimum temperature of the coldest month, annual precipitation and maximum temperature of the warmest month.

### MRC distribution under climate change

**Fig 2:**
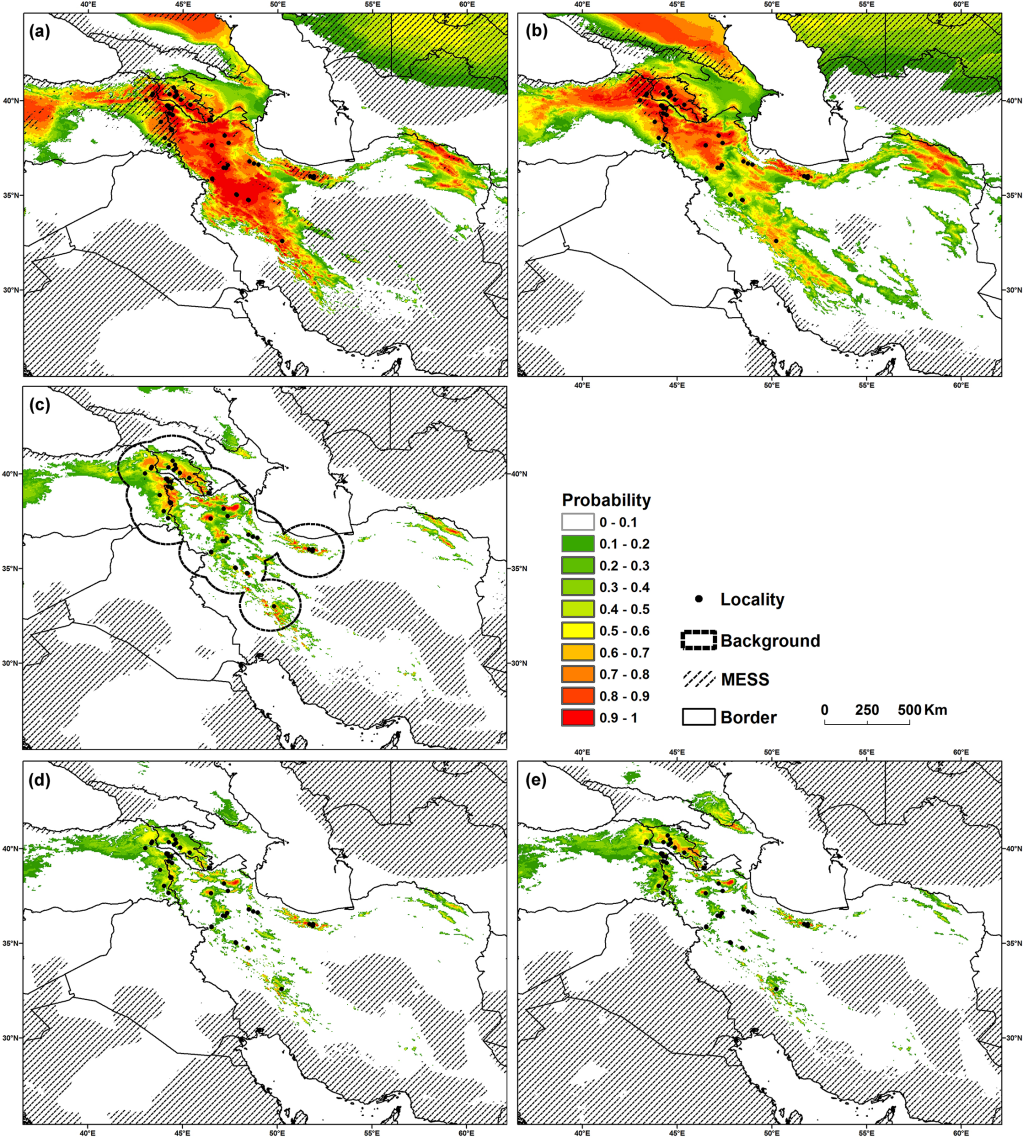
Predicted distribution models of *Montivipera raddei* species complex in Iran, Turkey and Armenia. Models are based on the CCSM (a) and MIROC (b) for the past, present condition (c), and 2.6 (d) and 8.5 (e) scenarios of CCSM that describe mitigation and high emission scenarios of future anthropogenic climate change, respectively. Red colour shows areas with higher probability of occurrence. MESS encompasses non-analogous climate conditions with reference to the training background range of the baseline model.

According to the results of the climate change models, the predicted past, current and future ranges of habitat of MRC are likely to be negatively affected by climate change scenarios (Fig 2). We found that, from the past to the future, MRC have experienced notable declines in the area of suitable habitats. With the expansion of unsuitable and less suitable climatic niches (probability class of 0–0.33) from 93.12% to 95.69% of the whole study area, the highly suitable climatic niche (probability class of 0.33–0.66) will be narrowed with accordance to future climate conditions (Table 3).

**Table 3:** Percentage cover of climate-only models within the probability classes for the distribution of *Montivipera raddei* species complex in Iran, Turkey and Armenia.

Probability classes	Past_CCSM	Past_MIROC	Current	Future_2.6	Future_8.5
0–0.33	77.12	77.75	92.66	96.69	97. 06
0.33–0.66	14.25	15.17	4.74	2.31	2.10
0.66–1	8.62	6.92	2.6	1	0.84

**Fig 3:**
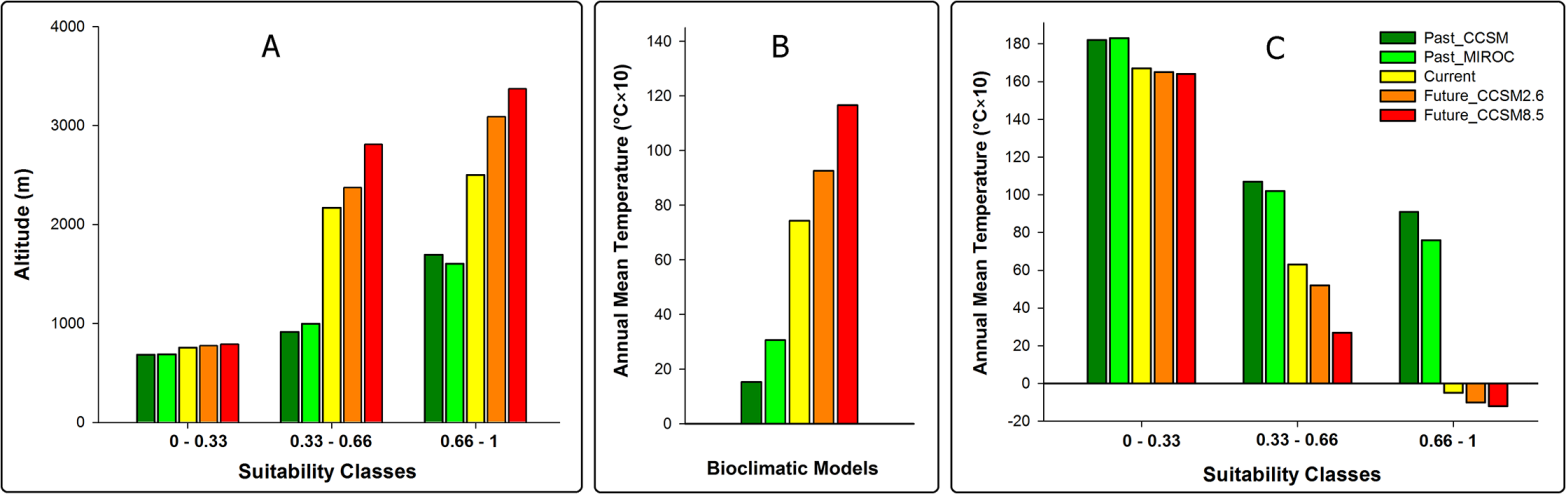
Altitudinal distribution of *Montivipera raddei* species complex habitats in the past, present and future (A). Annual mean temperature of accessible habitats for *Montivipera raddei* species complex within different climate niche models (B). Annual mean temperature of *Montivipera raddei* species complex habitats in the past, present and future (C). Average values were calculated for cells occurring within probability classes of different climate niche models.

After confirming changes in the bioclimatic niche of MRC, we tested our hypothesis of upward altitudinal shift by tracing the changes of average altitude within probability classes of climatic niche models. As we expected, our results revealed that average altitude within probability classes is higher for future scenarios in comparison with past and current conditions (Fig 3A). By comparing the mean annual temperature of accessible habitats we also found that under climate change scenarios, background space for MRC will shift from cool climate conditions to warmer conditions from the past to the future (Fig 3B) in concordance with the expected influence of global warming. However, under climate change scenarios modeling, our models revealed that MRC will experience a climate niche shift from warmer to colder conditions from the past to the future (Fig 3C).

## Discussion

### Present distribution

Allopatric populations of the MRC are mostly located within a matrix of presumably unsuitable habitat, indicating characteristics of relict and highly threatened populations [51]. As we had initially hypothesized, our results showed that there are various unidentified populations of *Montivipera* species across the Zagros, Alborz and Kopet Dagh Mountains in Iran and Ararat and lesser Caucasus mountains in Turkey and Armenia. Currently, Lar National Park in central Alborz, home to *Montivipera latifii* [15], is the westernmost established occurrence location of MRC [12]. However, our model indicated that Kopet Dagh Mountains in the northeast of Iran, some 700 km away from Lar National Park, contain suitable habitats for this species complex. Although this could be due to modeling weakness, we think it is possible that MRC populations occur in this region, but have not been identified due to poor sampling. Confirming the occurrence of new populations within the locations predicted by the model requires intensive field work. In fact, our visits to two of these habitat patches (Alvand and Bozgoush regions in the west and northwest of Iran, respectively) resulted in sighting MRC individuals and thus confirming the model’s predictive power. Some individuals found in the newly identified localities showed distinctive morphological characteristics, indicating that MRC populations have undergone unique local adaptations.

As expected, historical biogeography of MRC plays a major role in defining the current distribution of this species complex, with altitude identified as the variable that best explains the patchy distribution pattern. In general, apart from topographic and climatic variables, vegetation-related factors contributed substantially to explaining the current distribution of MRC. Vegetation preferred by MRC is mostly monotonous vegetation of pastures above tree-line and artificial forests in mountains [11] as indicated in our analyzes.

### Distribution contraction

*Montivipera raddei* species complex has faced continuous habitat contractions throughout its biogeographic history starting from thousands of years ago and continuing well into the future. Modeling the distribution of MRC in the past suggested that the gradual rise in temperature in most of the Iranian plateau after the last Maximal Glacial period [52] forced these species to move to higher altitudes in search for suitable habitats. However our findings reveal that the average temperature of suitable habitats of MRC will be colder in the future compared to the past (Fig 3C). Although this finding seems to contradict the expected influence of climate change, it can be explained as a probable shift in MRC’s habitat and thermal preference. Our findings suggest that in the past, MRC species occupied habitats in lower elevations, even in the bottom of valleys. At that time the higher elevations of the mountains were mostly covered in ice and snow. After the Last Glacial Maximum however, when temperatures increased in MRC's habitat, these species were bound to move to higher elevations (Fig 3A) to seek new habitats. This was in fact their only choice in order to survive. Our niche modeling indicates that although the overall average temperature of the Earth was rising, the local temperature in the new habitats of MRC located in higher elevation was probably lower than MRC’s previous habitats. In a 21000-year time frame, these vipers were able to adapt their thermal preference with the new thermal conditions. This finding is in concordance with the findings of Araujo *et al*. who stated “evolution of tolerances of terrestrial organisms to cold temperature should be more frequent than the evolution of high temperatures” [53].The upward shift in distribution of MRC has leads to a reduction in the total area of suitable habitat (Table 3), consequently isolating the populations in an even more severe patchy distribution (Fig 2).

Although our results clearly indicate that the present distribution of MRC can be related to the response of these species to climate change in the last 21000 years and that alterations in their niche is a response to the general trend of climate change, other factors could have contributed to the movement of these species from lowlands to high elevations. A possible alternative explanation for the upward shift from warmer to colder habitats is competition with other species such as *Macrovipera lebetina* that occupy lower elevations within the distribution range of MRC. It has been proposed that the presence of this species may have led to upward movement of MRC species [30]. Evidence supporting this hypothesis is provided by the geographic distribution and parapatric occurrence of *Macrovipera* and *Montivipera* in much of their range, but locally, as in parts of Armenia and eastern Turkey, *Macrovipera lebetina* and MRC are sympatric from 1200 m up to 2000 m asl. [11].

A second alternative explanation concerns anthropogenic activities in the lowland that could have caused local extinctions in MRC populations, leading the remaining populations to move to higher elevations, consequently causing their climatic niche to become colder than the past. However, not all lowland habitats at the distribution range of MRC have experienced alterations by human activities such as agriculture, ruling out this explanation as a prevalent cause of shift in MRC habitat suitability. Some important regions in distribution of MRC with unaltered lowland habitats include Amir-Abad (Takab), Lar National Park and Makoo in Iran and Khosrov and Shikahokh forest reserves in Armenia. The most important anthropogenic effects on MRC are in fact taking a toll on the species today. Illegal collection and overgrazing in the current distribution range takes place in the high elevations and can threaten long-term survival of these species.

Our models for the future distribution of MRC predicted a further upward shift in distribution of suitable habitats until 2070, leading to significant habitat loss due to global climate warming. Spatial fingerprints of climate change are usually associated with changes in the distribution of species at latitudinal or altitudinal extremes (see Results in [2,54–56]). Such altitudinal contractions in the distribution of mountain-dwelling reptiles have been established before (see Results in [57]) and the models that we developed verified our hypothesis that this is also true for MRC. Moreover, the possible reduction in the area of suitable habitats for MRC will lead to the local extinction of populations, leading to a loss of genetic diversity in these species, similar to what Rödder and Schulte [58] have predicted for *Lacerta schreiberi*.

Integrating molecular studies with ecological niche modeling allows for better understanding the evolutionary scenarios and responses of these species to climate change [59]. We suggest further researches on habitat shifts in MRC to focus on molecular studies to complement the results of the niche modeling achieved in the present study.

### Conservation biogeography

Overlapping the distribution map for MRC with the network of protected areas in the region showed that 10% of suitable habitats are being protected. This seems to be an acceptable coverage of the distribution range for these species. It must be noted that due to the cryptic nature of MRC, their presence in many of the protected areas in the region is not yet known. Communicating our results with the managers of such protected areas will play an important role in the conservation of these species. After confirming the presence of MRC populations within their managed areas, protected area managers will then be able to consider the critical habitats of these species within their management planning.

It is of paramount importance for the conservation of MRC to adopt an adaptive approach to reconsider the network of protected areas in the region. Establishing additional reserves based on suitable habitats identified in our models can considerably increase the effectiveness of MRC conservation. Moreover, according to the distribution pattern of reptiles in the mountainous zones of the Iranian plateau, the suitable habitats that we identified overlap with the distribution of syntopic species such as *Iranolacerta* genus [10] which are endemic to Iran. Conservation of the suitable MRC habitats will therefore yield positive outcomes for the conservation of other groups of reptiles as well.

Establishment of new protected areas, although necessary, will not ensure the viability of MRC populations, unless effective management would be practiced. The vegetative structure of MRC habitats makes them attractive to nomadic herders, exposing these valuable habitats to overgrazing [11]. Such human-caused habitat destruction exacerbates the deleterious effects of climate change [60]. Moreover, presence of herders and tourists in these habitats brings MRC in danger of illegal collection or killing. Therefore, apart from ensuring legal protection status for the MRC habitats, it is vital to promote and monitor effective protection of habitats and individuals within these reserves.

## Supporting Information

S1 Table Estimated relative contribution of eco-geographic variables to the general distribution model of *Montivipera raddei* species complex in Iran, Turkey and Armenia, calculated based on Maxent model. (DOCX)
