# Supplementary material for: Upward Altitudinal Shifts in Habitat Suitability of Mountain Vipers since the Last Glacial Maximum
Source: PLoS One. 2015 Sep 14;10(9):e0138087. doi: 10.1371/journal.pone.0138087 (PMC4569082; doi:10.1371/journal.pone.0138087)
Supplement: S1 Table — (DOCX) [file pone.0138087.s001.docx]

**S1 Table.** Estimated relative contribution of eco-geographic variables to the general distribution model of *Montivipera raddei* species complex in Iran, Turkey and Armenia, calculated based on Maxent model.

| **Percent contribution** | **Description (abbreviation)** | **Variable** |
| --- | --- | --- |
| 17.1 | Altitude: Elevation above sea level (altitude) | Topographic |
| 3.8 | Slope steepness (slope) |  |
| 1.9 | Solar Radiation Index (sri) |  |
| 1.6 | Distance to rainfed croplands (crop_dis) | Cover |
| 1.2 | Distance to mosaic vegetation/cropland (crop_veg_dis) |  |
| 1.4 | Distance to broadleaved deciduous forest /woodland (forest_dis) |  |
| 1.7 | Distance to mosaic forest or shrubland / grassland (frst_shrb_dis) |  |
| 10.5 | Distance to mosaic grassland (50-70%) / forest or shrubland (gras_shrb_dis) |  |
| 16.6 | Distance to closed to open (>15%) shrubland (shrub_dis) |  |
| 0.5 | Distance to closed to open herbaceous vegetation (herb_dis) |  |
| 1.3 | Distance to sparse (<15%) vegetation (sprs_veg_dis) |  |
| 3.1 | Annual precipitation (anulprc) | Bioclimatic |
| 1.1 | Precipitation in the driest month (driest) |  |
| 0.4 | Precipitation in the wettest month (wettest) |  |
| 0.0 | Precipitation seasonality (prcseas) |  |
| 14.3 | Annual mean temperature (anulmeantmp) |  |
| 2.2 | Minimum temperature of coldest month (coldest) |  |
| 0.0 | Maximum temperature of warmest month (warmest) |  |
| 0.8 | Temperature seasonality (tmpseas) |  |
| 1.8 | Mean temperature of May (tmean5) |  |
| 1.5 | Maximum temperature of May (tmax5) |  |
| 14.4 | Minimum temperature of May (tmin5) |  |
| 2.8 | Precipitation in May (prc5) |  |
